# Distribution of resistance genes encoding ESBLs in *Enterobacteriaceae* isolated from biological samples in health centers in Ouagadougou, Burkina Faso

**DOI:** 10.1186/s13104-018-3581-5

**Published:** 2018-07-13

**Authors:** Dissinviel S. Kpoda, Abraham Ajayi, Marius Somda, Oumar Traore, Nathalie Guessennd, Aboubakar S. Ouattara, Lassana Sangare, Alfred S. Traore, Mireille Dosso

**Affiliations:** 1 Laboratoire des Sciences Appliquées et Nutritionnelles (LabSAN), Centre de Recherche en Sciences Biologiques, Alimentaires et Nutritionnelles (CRSBAN), Université Ouaga 1 Pr Joseph KI-ZERBO, 03 BP 7021, Ouagadougou 03, Burkina Faso; 2 Laboratoire National de Santé Publique, 09 BP 24, Ouagadougou 09, Burkina Faso; 3 Centre Hospitalier Universitaire Yalgado Ouedraogo, 03 BP 7022, Ouagadougou 03, Burkina Faso; 4 Département de Bactériologie et de Virologie, Institut Pasteur de Côte d’Ivoire, 01 BP 490, Abidjan 01, Côte d’Ivoire; 5 Department of Microbiology, University of Lagos, Akoka, Nigeria; 6 Unité de Formation et de Recherche en Sciences Appliquées et Technologiques (UFR/SAT)/Institut des Sciences de l’Environnement et du Développement Rural (ISEDR), Centre Universitaire Polytechnique de Dédougou, BP 07, Dédougou, Burkina Faso

**Keywords:** Extended spectrum beta-lactamase, *bla*-*TEM*, *bla*-*SHV*, *bla*-*CTX*-*M*, Burkina Faso

## Abstract

**Objective:**

Resistance to antibiotics most especially third generation cephalosporins has assumed a worrisome dimension globally. Genes conferring these resistance which are mediated by enzymes known as extended spectrum beta-lactamases (ESBLs) are now wide spread among several *Enterobacteriaceae* species. However there is paucity of data regarding the distribution of these genes in Burkina Faso. Hence this prospective study aims to determine the prevalence and distribution of ESBL encoding genes in ESBL producing *Enterobacteriaceae* strains isolated from clinical samples of patients attending the three major hospitals in Ouagadougou Burkina Faso.

**Results:**

ESBL-encoding genes were assayed in 187 ESBL producing *Enterobacteriaceae* strains. Among these isolates, the prevalence of ESBL-producing strains with bla*TEM*, bla*SHV* and bla*CTX*-*M* genes were 26.2% (49/187), 5.9% (11/187) and 40.1% (75/187) respectively. The association of ESBL encoding genes with health centers was statistically significant (p = 0.0209). Approximately 39.6% of *E. coli* harbored *CTX*-*M* and *Klebsiella* spp. 5.9%. This study demonstrates the dissemination of *TEM*, *SHV* and *CTX*-*M* genes in ESBL producing *Enterobacteriaceae* strains in Ouagadougou. Continuous spread of these bacteria poses great public health risk, thus increased surveillance and regulation of antibiotics use is imperative in Burkina Faso.

## Introduction

The problem of microbial drug resistance has assumed a global dimension and an alarming magnitude, being one of the leading unresolved problems in public health [1]. Beta-lactam antibiotics (penicillins, cephalosporins, monobactams and carbapenems) are among the most prescribed drugs in the world [2]. Their use is followed by resistance observed in several *Enterobacteriaceae* species [3]. Mechanism of resistance includes: topoisomerase mutation by reducing antibiotic affinity, activation of efflux system which expels antibiotics out of the cell and antibiotic inactivation by enzymes [4, 5]. A key mechanism of note is the production of β-lactamase that hydrolyzes the β-lactam ring [6, 7]. Broad spectrum beta-lactamases are enzymes that have the particularity to hydrolyze all beta-lactams including third-generation cephalosporins except cephamycins and carbapenems [8, 9]. The number of extended-spectrum beta-lactamases producing *Enterobacteriaceae* isolates (ESBL) has been on the increase in recent times worldwide [10]. Several antibiotic resistance genes studies have been carried out in Burkina Faso [11–14], but they are all restricted to single health centers in Ouagadougou city. Hence the aim of this study is to determine the prevalence and distribution of resistance genes coding for broad spectrum beta-lactamases in the three major health centers of Ouagadougou, Burkina Faso. Since hospitals in recent years has been considered as reservoirs of ESBL producing *Enterobacteriaceae* [15].

## Main text

Bacterial strains consisted of isolates obtained from our previous report [16]. They were obtained from patients attending three major hospitals Yalgado Ouedraogo Teaching Hospital (CHU-YO), Charles De Gaulle Paediatric Teaching Hospital (CHUP-CDG) and Saint Camille Hospital (HOSCO) in Ouagadougou Burkina Faso. Isolates were obtained from urine samples, pus samples, blood samples, stool samples, vaginal swab samples and pleural fluid samples.

### Methodology

Genomic DNA was extracted according to Guedda et al. [17] with slight modification. One to five bacterial colonies were suspended in 100 ml of nuclease free water and suspension was heated at 100 °C for 10 min. After centrifugation at 12,000×*g* for 10 min at 4 °C, 5 µL of supernatant were used as template in a 50 µL PCR reaction [18]. Resistance genes *blaTEM*, *blaCTX*-*M*, *blaSHV* were detected by PCR. PCR was performed in a final volume of 50 μL using the set of primers shown in Table 1. The reaction mix consist of 5× colored buffer 5 μL and 5× unshaded buffer, 3 μL of MgCl_2_ (25 mM) (Promega, USA), 0.5 μL of dNTP (10 mM), 0.5 μL of each primer (20 mM) (Sigma Genesys) and 0.2 μL of Taq polymerase (GoTaq^®^ G2 Flexi DNA polymerase, USA, Reference M7805) with a volume of 5 μL of DNA. PCR amplification conditions of 30 cycles of initial denaturation 94 °C for 5 min, denaturation 94 °C for 1 min, annealing at 50 °C for 1 min (bla-TEM) and 60 °C for 1 min (bla-SHV and bla-CTX-M), elongation 72 °C for 1 min and final elongation for 72 °C for 7 min were carried out in a thermal cycler (GeneAmp^®^ Applied Biosystem). Amplicons were electrophoresed on 1.5% agarose gel containing TAE buffer at 135 V for 30 min (90 mMTris, 90 mM acetate, 2 mM EDTA, pH 8.0) (TAE Buffer, USA) with DNA Ladder 1 kb (Promega, USA). Four bacteria strains were used as positive controls *Salmonella* spp. (U2A1446) for *bla*-*TEM* and *bla*-*SHV*, *E. coli* (U2A1790) for *bla*-*CTX*-*M* group 1, *E. coli* (U2A1799) for *bla*-*CTX*-*M* group 2 and *E. coli* (U2A1796) for *bla*-*CTX*-*M*-9 and DNA free reaction mixture as negative controls. Data obtained were entered and analyzed using Excel and GraphPad Prism version 5.01 software. A p-value less than 0.05 was considered to be statistically significant (p < 0.05).

**Table 1:** Sequences of primers used

Genes	Primers	Sequence (5′–3′)	Weight (pb)	Accession number
bla*tem*	a 216 (+)	ATAAAATTCTTGAAGACGAAA	1079	AB282997
	a 217 (−)	GACAGTTACCAATGCTTAATCA		
bla*shv*	os-5 (+)	ATTTGTCGCTTCTTTACTCGC	1051	X98098
	os-6 (−)	TTTATGGCGTTACCTTTGACC		
bla*ctx*-*M*	ctxM1 (+)	GGTTAAAAAATCACTGCGTC	863	X92506
	ctxM1 (−)	TTGGTGACGATTTTAGCCGC		
	ctxM2 (+)	ATGATGACTCAGAGCATTCG	865	X92507
	ctxM2 (−)	TGGGTTACGATTTTCGCCGC		
	ctxM9 (+)	ATGGTGACAAAGAGAGTGCA	869	AF174129
	ctxM9 (−)	CCCTTCGGCGATGATTCTC		

### Results

**Fig. 1:**
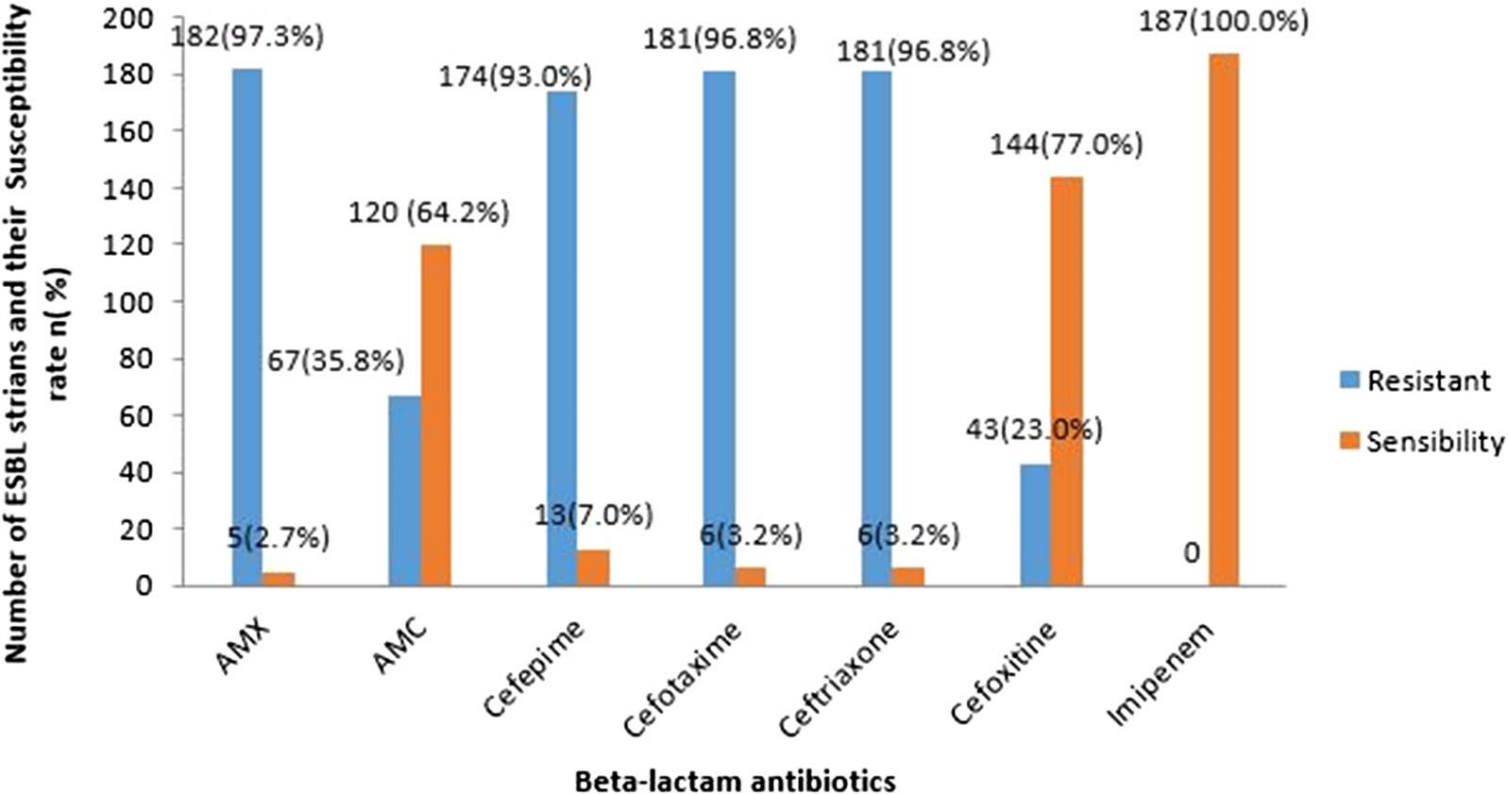
ESBL strains susceptibility to beta-lactam antibiotics. *AMX* amoxicillin, *AMC* amoxicillin + clavulanic acid7

Phenotypic profile of ESBL producing isolates is shown in Fig. 1. A combination of resistance genes from various bacterial species were observed as shown in Table 2. The occurrence of blaCTX-M gene, *blaTEM* gene and *blaSHV* gene in *E. coli* was 39.6, 24.6 and 3.7% respectively compared to *Klebsiella* spp. that had 5.9% *blaCTX*-*M* gene, 2.7% *blaTEM* and 1.6% *blaSHV* gene occurrence. *blaTEM*-1 gene was found in all 3 health centers whereas CTX-M-9 was detected in bacteria isolated from CHU-YO. *blaSHV*-1, *blaCTX*-*M*-*1* and *bla*CTX-M-2 genes were detected in bacteria isolated from CHU-YO and CHUP-CDG. *blaCTX*-*M*-9 gene was detected in *Proteus* sp. strain isolated from CHU-YO. However, a combination of 3 *bla* genes were detected in only isolates from CHU-YO and two bla genes were detected in isolates from CHU-YO and CHUP-CDG. Association of ESBL encoding genes with health centers was significant (p = 0.0209).

**Table 2:** Distribution of resistance genes *bla*TEM, *bla*SHV, *bla*CTX-M according to the health center

*Enterobacteriaceae* strains (n/N)	Resistance genes
Yalgado Ouedraogo University Hospital (CHU-YO)	
*K. pneumoniae* (1/5)	TEM-1
*K. pneumoniae* (2/5)	SHV-1
*K. pneumoniae* (1/5)	CTX-M-1
*K. pneumoniae* (1/5)	CTX-M-2
*K. pneumoniae* (1/5)	TEM-1 + CTX-M-1
* K. pneumoniae* (1/5)	TEM-1 + CTX-M-2
*K. pneumoniae* (1/5)	CTX-M-1 +CTX-M-2
*K. pneumoniae* (1/5)	TEM-1 + CTX-M-1 + CTX-M-2
*K. oxytoca* (1/1)	SHV-1
*Klebsiella sp.* (5/19)	CTX-M-1
*E. coli* (4/81)	TEM-1
*E. coli* (9/81)	CTX-M-1
*E. coli* (9/81)	CTX-M-2
*E. coli* (4/81)	TEM-1 + CTX-M-1
*E. coli* (4/81)	TEM-1 + CTX-M-2
*E. coli* (9/81)	CTX-M-1 + CTX-M-2
*E. coli* (4/81)	TEM-1 + CTX-M-1 + CTX-M-2
*Proteus sp*. (1/4)	SHV-1
*Proteus sp.* (1/4)	CTX-M-9
Pediatric Charles De Gaulle University Hospital (CHUP-CDG)	
*E. coli* (14/27)	TEM-1
*E. coli* (14/27)	CTX-M-1
*E. coli* (7/27)	CTX-M-2
*E. coli* (7/27)	SHV-1
*E. coli* (14/27)	TEM-1 + CTX-M-1
Saint Camille Hospital of Ouagadougou (HOSCO)	
*E. coli* (2/9)	TEM-1

### Discussion

The presence of ESBL-producing bacteria in hospitals poses a serious challenge. This challenge cuts across developed and developing countries. In a multicenter study carried out in 18 representative hospitals in France, it was revealed that ESBL encoding genes including CTX-M-15, CTX-M-1, CTX-M-14 and SHV-12 were most prevalent [19]. Hospitals in Burkina Faso are no exception to this fact. In our study two major *Enterobacteriaceae E. coli* and *Klebsiella* spp. bearing various ESBL encoding genes dominated in all three major hospitals in Ouagadougou. This concurs with the findings of Najjuka et al. [20] that reported high prevalence of ESBL-producing *E. coli* and *K. pneumoniae* isolated from clinical samples in Uganda. Mathlouthi et al. [21] reported the distribution of *blaCTX*-*M*-*15* (51.7%), *blaTEM*-*1* (35.6%) and variants of *blaSHV* (21.8%) in *Klebsiella* spp. and *E. coli* stains isolated from hospitals in Tunisia and Libya. This is similar to our findings, however with lower percentage occurrence of bla genes in both *E. coli* (39.6% *blaCTX*-*M*, 24.6% *blaTEM*, and 3.7% *blaSHV*) and *Klebsiella* spp. (5.9% *blaCTX*-*M*, 2.7% *blaTEM*, and 1.6% *blaSHV*). This does not make the challenge in treatment and management of infection caused by these bacteria in Burkina Faso any lesser. In a similar study in Burkina Faso Ouedraogo et al. [14] reported *CTX*-*M*-1 group as dominant followed by *CTX*-*M*-9 group, this corroborate our findings with *CTX*-*M*-1 and *CTX*-*M*-2 dominating in two of the hospitals (CHU-YO and CHUP-CDG). This is in line with the reports of Ibrahim et al., Cantón et al. and Poirel et al. [22–24] that, *bla*-*CTX*-*M* genes are the most common types of ESBL in microorganisms in most areas in the world while there seems to have been a drastic decrease of *blaSHV*. These identified genes play a major role in conferring resistance to extended-spectrum cephalosporins and other β-lactam antibiotics as exemplified in the results of our study. Ninety-three percent (93%) of isolates were resistant to cefepime, 96.8% resistant to cefotaxime, 96.8% resistant to ceftriaxone and 23% resistant to cefoxitine. However no isolate was resistant to imipenem a carbapenem. This corroborates the findings of El bouamri et al. [25] who reported *E. coli* armed with ESBL encoding genes that were resistant to cefotaxime, ceftazidime, cefepime and other β-lactam antibiotics but none was resistant to imipenem. The significance (p < 0.05) of ESBL encoding genes in strains isolated from health centers implies that hospitals remain a key hub in the potential dissemination of ESBL-producing *Enterobacteriaceae*. This is in line with the findings of Ahmed et al. [26] that detected *bla*-*SHV* and *bla*-*CTX*-*M* genes in *Klebsiella pneumoniae* isolates from patients with suspected nosocomial infections in Egypt.

### Conclusions

The emergence, dissemination and expansion of ESBL- producing *Enterobacteriaceae* poses a serious public health challenges resulting in substantial limitations in the efficacy of therapeutic interventions. Hence prevention and proactive surveillance of antimicrobial resistance is crucial. Furthermore according to Zahar et al. [27] screening strategies should be established for early identification of patients that are carriers of ESBL producing bacteria in hospitals. Prescription of imipenem should be done with caution and monitored as it remains a viable option in a situation of overwhelming resistance of *Enterobacteriaceae* to other classes of routinely used antibiotics.

## Limitations

This study enrolled patients attending only hospitals located in Ouagadougou Burkina Faso. Future Study should cover other hospitals across the country to determine entire country wise prevalence.

## Abbreviations

ESBL: extended spectrum beta-lactamase
CHU-YO: Yalgado Ouedraogo University Hospital
CHUP-CDG: Pediatric Charles De Gaulle University Hospital
HOSCO: Saint Camille Hospital of Ouagadougou
